# Functional and neurometabolic asymmetry in SHR and WKY rats following vasoactive treatments

**DOI:** 10.1038/s41598-019-52658-9

**Published:** 2019-11-06

**Authors:** Ana B. Segarra, Isabel Prieto-Gomez, Inmaculada Banegas, Magdalena Martínez-Cañamero, Juan de Dios Luna, Marc de Gasparo, Manuel Ramírez-Sánchez

**Affiliations:** 1 Department of Health Sciences, University of Jaén, 23071 Jaén, Spain; 2 Department of Biostatistic, Medical School, University of Granada, Granada, Spain; 3 Cardiovascular & Metabolic Syndrome Adviser, Rue es Planches 5, 2842 Rossemaison, Switzerland

**Keywords:** Neuro-vascular interactions, Neurophysiology

## Abstract

A lateralized distribution of neuropeptidase activities in the frontal cortex of normotensive and hypertensive rats has been described depending on the use of some vasoactive drugs and linked to certain mood disorders. Asymmetrical neuroperipheral connections involving neuropeptidases from the left or right hemisphere and aminopeptidases from the heart or plasma have been suggested to play a role in this asymmetry. We hypothesize that such asymmetries could be extended to the connection between the brain and physiologic parameters and metabolic factors from plasma and urine. To assess this hypothesis, we analyzed the possible correlation between neuropeptidases from the left and right frontal cortex with peripheral parameters in normotensive (Wistar Kyoto [WKY]) rats and hypertensive rats (spontaneously hypertensive rats [SHR]) untreated or treated with vasoactive drugs such as captopril, propranolol and L-nitro-arginine methyl ester. Neuropeptidase activities from the frontal cortex were analyzed fluorometrically using arylamide derivatives as substrates. Physiological parameters and metabolic factors from plasma and urine were determined using routine laboratory techniques. Vasoactive drug treatments differentially modified the asymmetrical neuroperipheral pattern by changing the predominance of the correlations between peripheral parameters and central neuropeptidase activities of the left and right frontal cortex. The response pattern also differed between SHR and WKY rats. These results support an asymmetric integrative function of the organism and suggest the possibility of a different neurometabolic response coupled to particular mood disorders, depending on the selected vasoactive drug.

## Introduction

The analysis of the significance of cerebral lateralization in humans, understood as a morphological, functional or neurochemical difference between the left and right hemispheres as well as the interaction of both, is more than 100 years old. Lateralization remains, however, an ill-defined topic that is sometimes undervalued^1,2^. Brain asymmetry is also applicable to non human species including rodents^3^ in which lateralized behavioral functions were described^4^. Lateralized neurochemical relationships for an asymmetrical behavior in rats^5^ were also described in early research on rat brain asymmetry. Regarding the present study, the existence of brain asymmetry in rats for a neuropeptidase activity (leucyl-aminopeptidase) was initially described in frontal cortex^6^. Various sets of data confirm that asymmetry extends to all organizational levels of the main internal communication systems: the central and peripheral nervous system as well as endocrine glands^7^.

**Figure 1:**
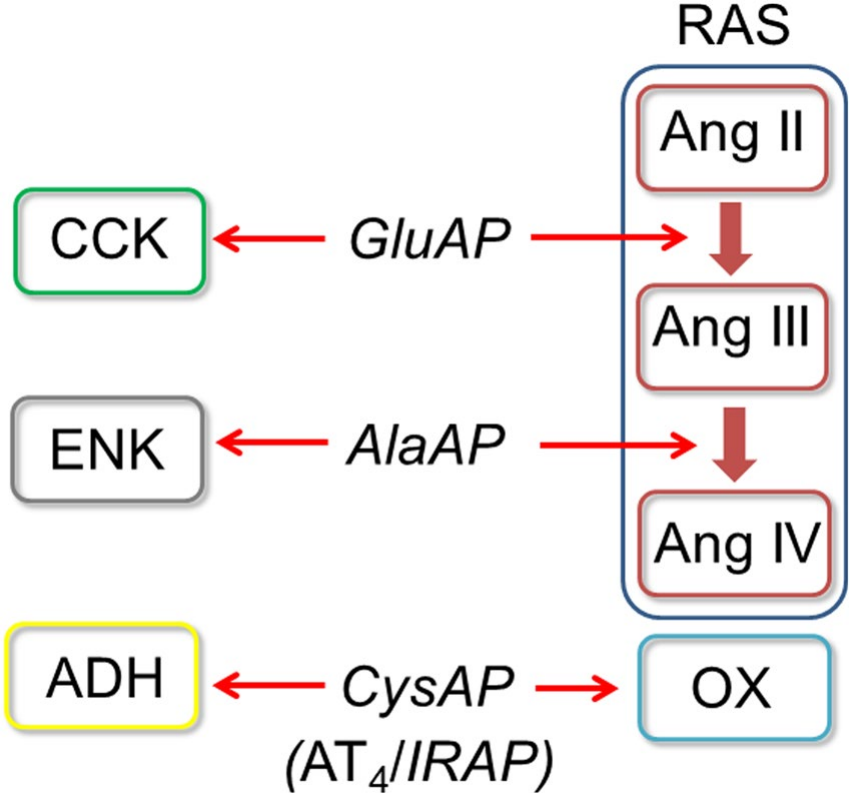
Potential endogenous substrates of the enzymatic activities analyzed in the present work. Glutamyl aminopeptidase (GluAP, EC 3.4.11.7)) metabolizes Ang II to produce Ang III and also hydrolyzes cholecystokinin (CCK)^16^. Alanyl aminopeptidase (AlaAP, EC 3.4.11.2) metabolizes Ang III to form Ang IV^13^ and also acts as enkephalinase^12^ regulating enkephalin (ENK) function. Cystinyl aminopeptidase (CysAP, EC 3.4.11.3) which has been reported to be the Ang IV receptor (AT_4_)^15^ or the insulin-regulated aminopeptidase (IRAP), involved in cell glucose uptake^15^, also acts as oxytocinase and vasopressinase^14^ regulating oxytocin (OX) and vasopressin (antidiuretic hormone, ADH). Partial steps of the enzymatic cascade of the renin-angiotensin system (RAS) are represented in the diagram.

An asymmetrical correlation between central neuropeptidases of the frontal cortex and peripheral peptidases from plasma^8^ and the heart^9^ has been reported in rats. We postulate that such a correlation may be the result of an integrative response of the organism and that this asymmetrical connection could be extended to a general concept of neurovisceral asymmetric integration. To support our proposal and to advance the functional understanding of this connection, we hypothesize that such asymmetry could be reflected in the pattern of behavior between the left and right brain and physiological parameters as well as metabolic factors present in plasma or urine. To test this hypothesis, we studied the existence of possible correlations between neuropeptidase activities in the left and right frontal cortex, a brain region involved in cognitive, physiologic and metabolic processes^10^, and physiological parameters such as water intake (WI), diuresis (DIU) and water balance (WB). We also investigated the correlations between these neuropeptidase activities from the left and right frontal cortex with some metabolic factors determined in plasma (such as high-density lipoprotein (HDL), low-density lipoprotein (LDL), HDL/LDL, glucose (GLU) and nitric oxide (NO) and urine (such as proteinuria, NO and creatinine). These studies were performed in nontreated (control (CT)) Wistar Kyoto (WKY) rats and spontaneously hypertensive rats (SHR) and in the same strains treated with vasoactive drugs such as the angiotensin-converting enzyme (ACE) inhibitor captopril (CAP), the beta-blocker propranolol (PRO) and the nitric oxide synthase inhibitor L-nitro-arginine methyl ester (L-NAME or LN). These vasoactive drugs were selected on the basis of their different mechanism of action on the cardiovascular system and their opposite influence on blood pressure^11^. The activities of three neuropeptidases, alanyl aminopeptidase (AlaAP), involved in enkephalin and angiotensin III metabolism^12,13^, cystinyl aminopeptidase (CysAP), involved in oxytocin and vasopressin metabolism^14^, also identified as the insulin-regulated aminopeptidase (IRAP) or the Ang IV receptor (AT_4_)^15^, and glutamyl aminopeptidase (GluAP), involved in angiotensin II and cholecystokinin metabolism^16^ (Fig. 1), were measured fluorometrically using arylamide derivatives as substrates^17^ as a reflection of the functional status of their endogenous substrates.

## Results

### Physiologic parameters and metabolic factors

The levels of neuropeptidase activities in the left and right frontal cortex as well as the blood pressure values for the groups of animals included in this study were previously reported^11^. All of the groups with SHR demonstrated higher blood pressure values (mmHg) (mean ± SEM: CT 206.7 ± 6.8, CAP 159.3 ± 5.2, PRO 206.7 ± 3.8, LN 234.7 ± 4.1) than the corresponding groups with WKY rats (mean ± SEM: CT 143.7 ± 4.5, CAP 121.8 ± 11.3, PRO 139 ± 5.8, LN 215 ± 2.6). The highest blood pressure levels were observed in the LN group and the lowest in the CAP group in both WKY rats and SHR. No significant differences were observed between the CT and PRO groups in WKY rats and SHR^11^.

**Figure 2:**
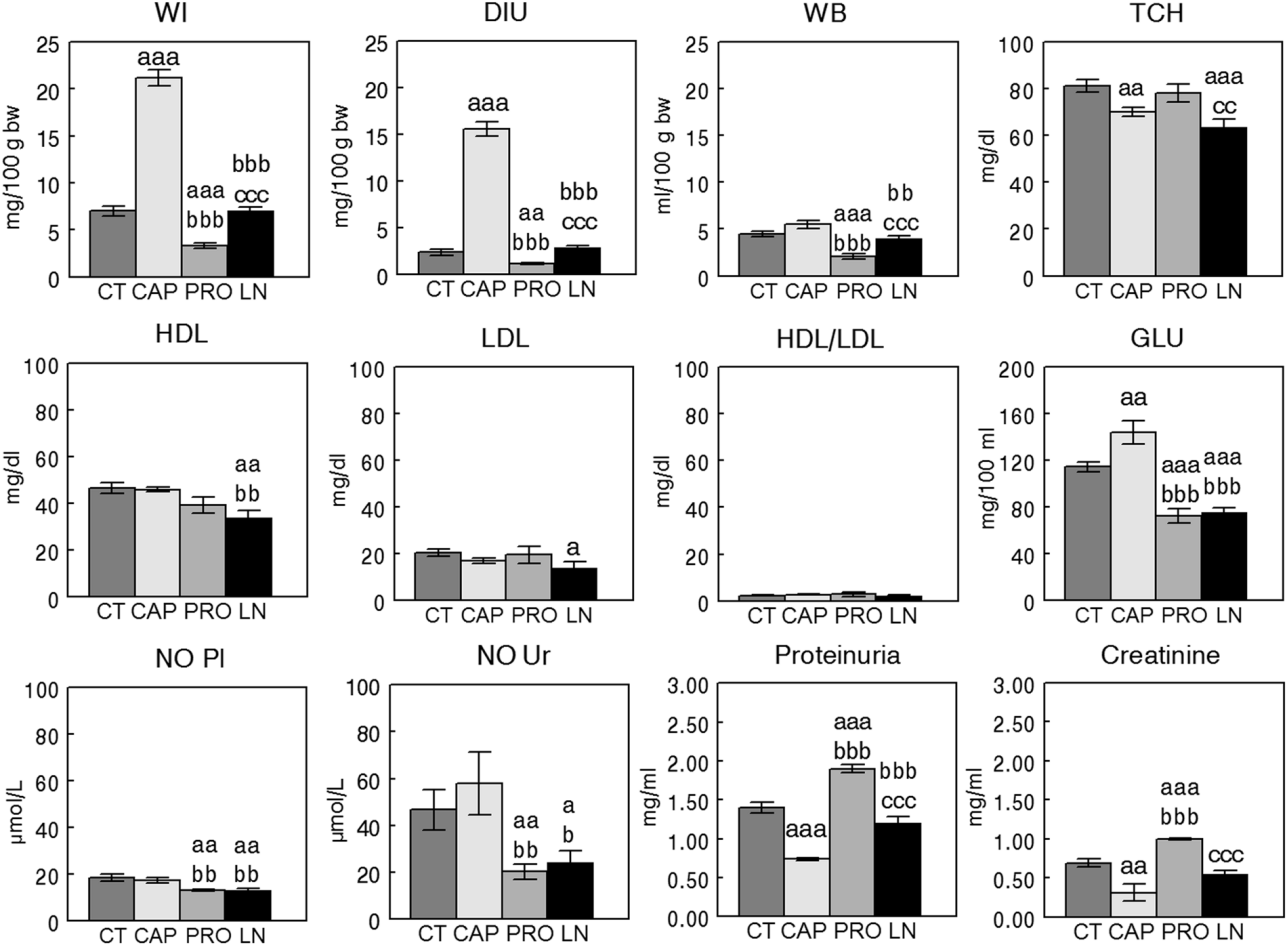
Mean ± SEM levels of the different physiologic parameters and metabolic factors measured in plasma and urine of Wistar Kyoto (WKY) rats (n = 8). Control (CT), captopril (CAP), propranolol (PRO) L-NAME (LN). (**a**) Comparison vs CT, (**b**) vs CAP, (**c**) vs PRO; single letter p < 0.05, double letter p < 0.01, triple letter p < 0.001. WI, water intake; DIU, diuresis; WB, water balance; TCH, total cholesterol; HDL, high-density lipoprotein; LDL, low-density lipoprotein; GLU, glucose; NO Pl, nitric oxide plasma; NO Ur, nitric oxide urine.

**Figure 3:**
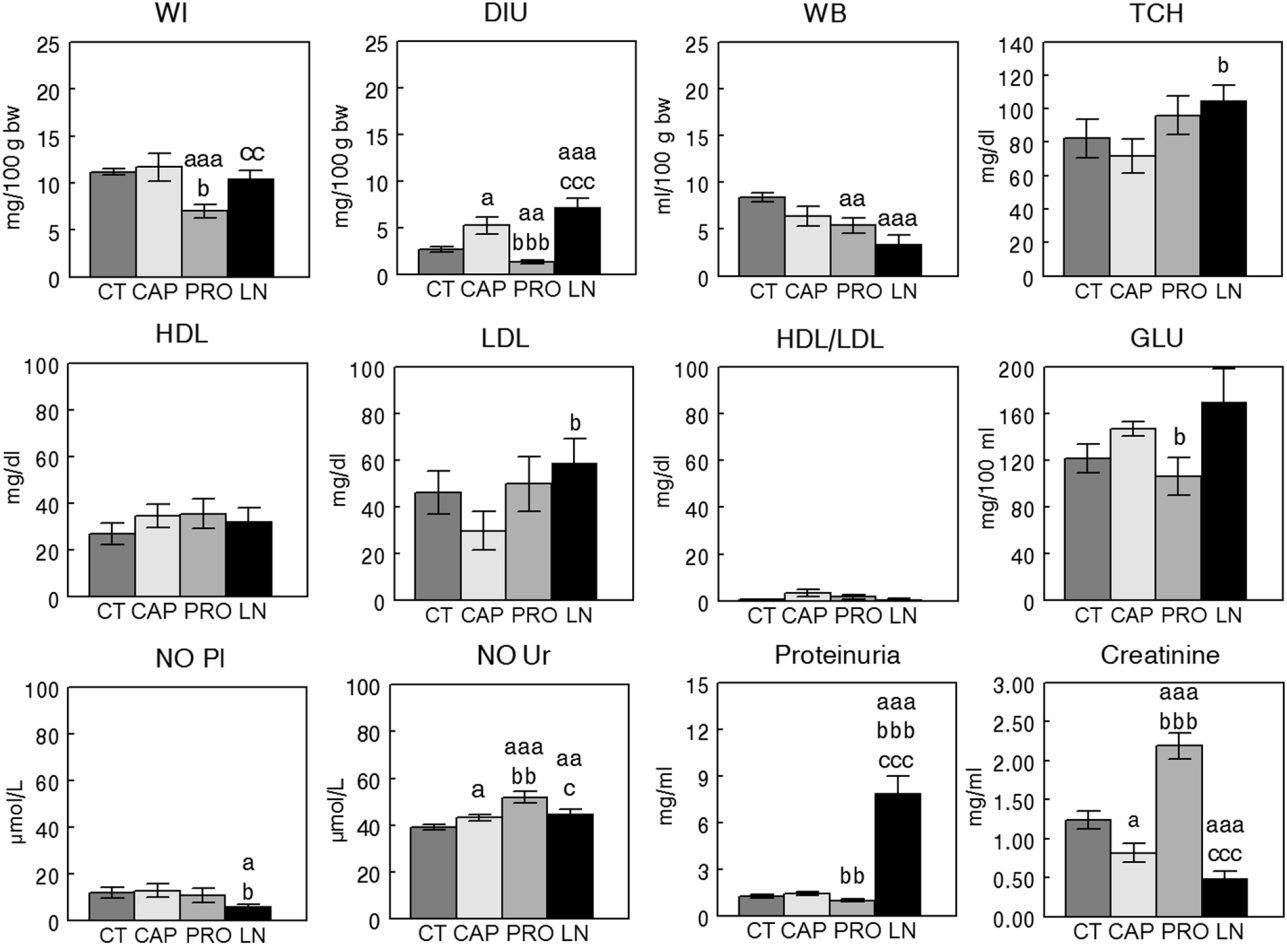
Mean ± SEM levels of the different physiologic parameters and metabolic factors measured in plasma and urine of spontaneously hypertensive rats (SHR) (n = 8). Control (CT), captopril (CAP), propranolol (PRO) L-NAME (LN). (**a**) Comparison vs CT, (**b**) vs CAP, (**c**) vs PRO; single letter p < 0.05, double letter p < 0.01, triple letter p < 0.001. WI, water intake; DIU, diuresis; WB, water balance; TCH, total cholesterol; HDL, high-density lipoprotein; LDL, low-density lipoprotein; GLU, glucose; NO Pl, nitric oxide plasma; NO Ur, nitric oxide urine.

Figures 2 and 3 represent the mean ± SEM values of the different physiologic and metabolic parameters studied and the statistical significance of their comparisons, respectively, in the groups with SHR or WKY rats.

In WKY rats (Fig. 2), the CAP group exhibited the highest WI and DIU values, whereas the PRO group exhibited the lowest. While CT and CAP groups did not differ between them for WB, the PRO group demonstrated the lowest levels of WB amongst groups. TCH levels were significantly lower in the CAP and LN groups than in the CT.

HDL in the LN group was lower than that in the CT and CAP groups, and LDL in the LN group was lower than that in the CT group. No differences between groups were observed for HDL/LDL values. GLU was higher in the CAP group than in the other groups and lower in the PRO and LN groups than in the CT and CAP groups. NO was lower in the PRO and LN groups than in the CT and CAP groups in both plasma and urine. The lowest levels of proteins and creatinine in urine were observed in the CAP group and the highest in the PRO group.

In SHR (Fig. 3), WI and DIU were lower in the PRO group than in the other groups. Furthermore, WB was lower in the PRO and LN groups than in the CT group. TCH was higher in the LN group than in the CAP group. While no differences between groups were observed for HDL and HDL/LDL, LDL was higher in the LN group than in the CAP group. GLU was lower in the PRO group than in the CAP group, and plasma NO was lower in the LN group than in the CT and CAP groups. In urine, NO was lower in the CT group than in the other groups. The levels of proteinuria were highest in the LN group, and the levels of creatinine in urine were higher in the PRO group than in the other groups.

**Figure 4:**
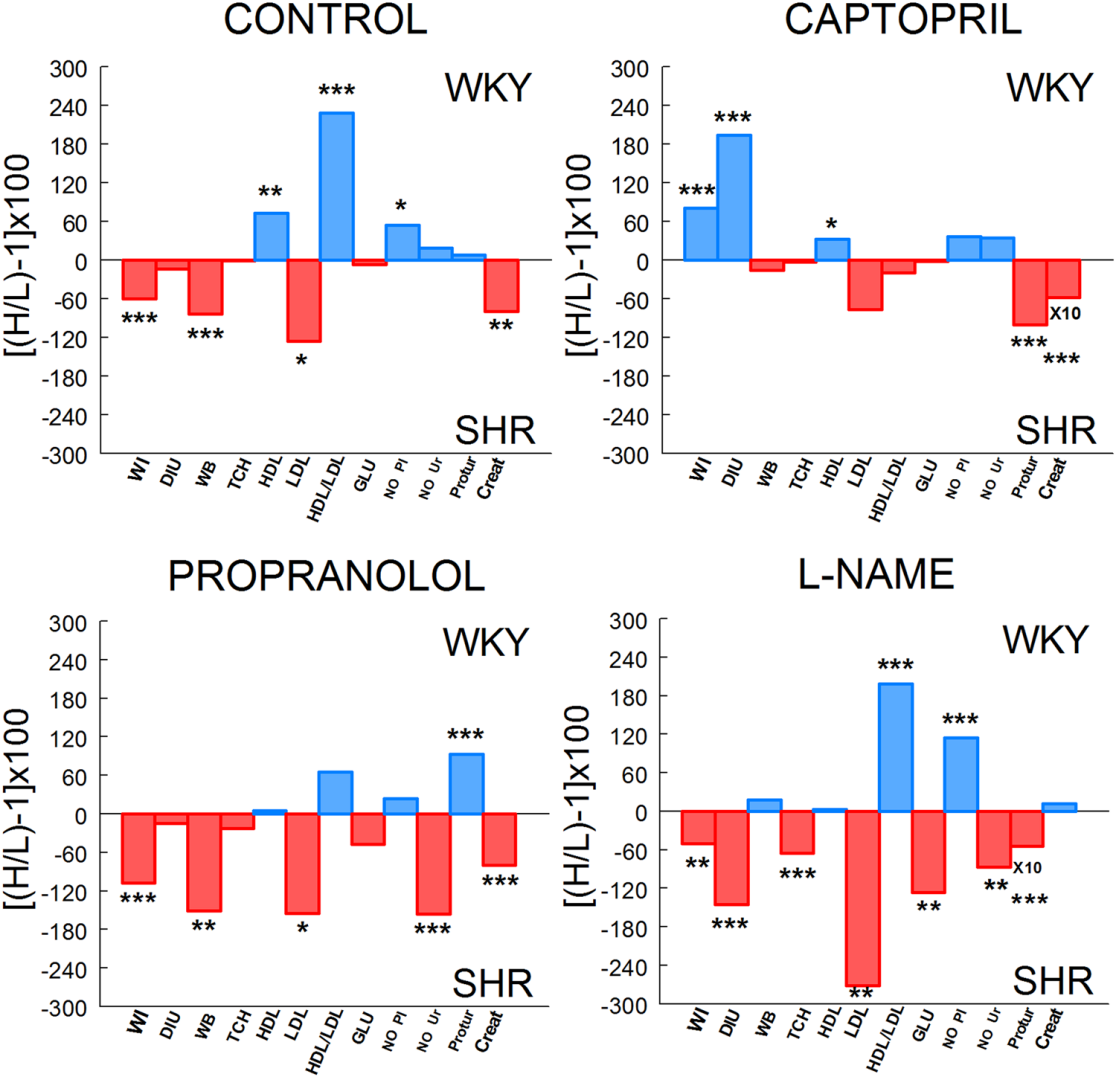
Comparisons of functional and metabolic parameters between WKY and SHR. Bars indicate the percentages of predominance of each parameter in WKY or SHR obtained from mean values of both strains in control, captopril, propranolol and L-NAME treated groups. Positive (blue) values indicate predominance in WKY. Negative (red) values indicate predominance in SHR. Values of creatinine (Creat) in captopril group and proteinuria (Protur) in the L-NAME group are ten folds (X10) higher. Asterisks indicate the level of significance when the corresponding parameter was compared between WKY and SHR. H, higher mean value; L, lower mean value. *P < 0.05, **P < 0.01; ***P < 0.001.

Comparisons of functional and metabolic parameters between SHR and WKY rats are illustrated in Fig. 4. In controls, SHR demonstrated higher values than WKY for WI (*P* < 0.001), WB (*P* < 0.001), LDL (*P* < 0.05) and creatinine (*P* < 0.01). In contrast, HDL (*P* < 0.01), HDL/LDL (*P* < 0.001) and NO Pl (*P* < 0.05) predominated significantly in WKY. In captopril treated animals, WI (*P* < 0.001), DIU (*P* < 0.001) and HDL (*P* < 0.05) predominated significantly in WKY whereas proteinuria (*P* < 0.001) and creatinine (*P* < 0.001) prevailed in SHR. In propranolol treated rats, while WI (*P* < 0.001), WB (*P* < 0.01), LDL (*P* < 0.05), NO Ur (*P* < 0.001) and creatinine (*P* < 0.001) predominated in SHR, only proteinuria (*P* < 0.001) prevailed significantly in WKY. In the L-NAME group, WI (*P* < 0.01), DIU (*P* < 0.001), TCH (*P* < 0.001), LDL (*P* < 0.01), GLU (*P* < 0.01), NO Ur (*P* < 0.01) and proteinuria (*P* < 0.001) predominated in SHR whereas HDL/LDL (*P* < 0.001) and NO Pl (*P* < 0.001) predominated in WKY.

### Correlational study

**Figure 5:**
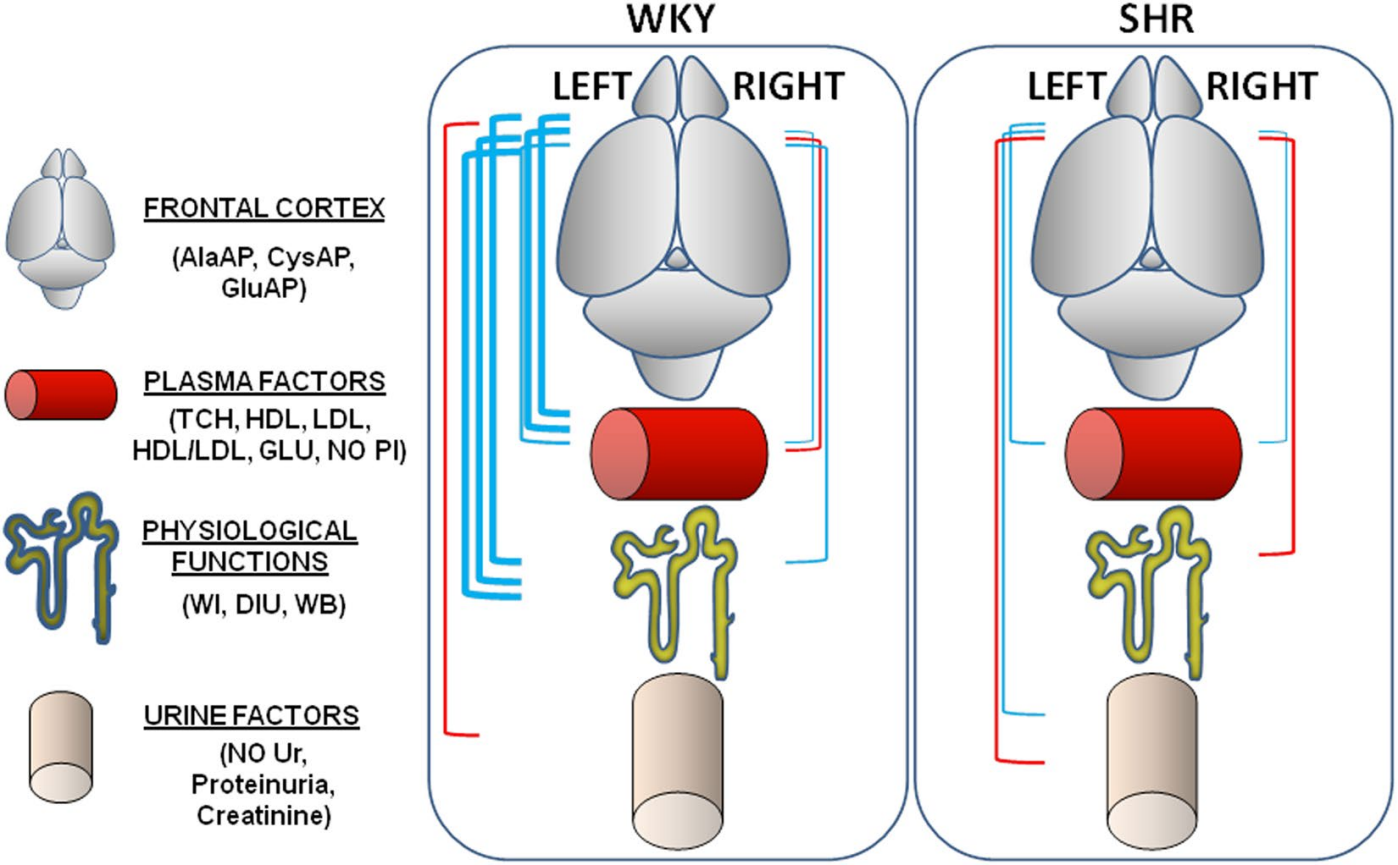
Schematic representation of all the significant positive (blue lines) and negative (red lines) correlations between neuropeptidase activities (AlaAP, CysAP, GluAP) of the left and right frontal cortex with physiologic parameters (WI, DIU, WB), plasma factors (TCH, HDL, LDL, HDL/LDL, GLU, NO Pl) and urine metabolites (NO Ur, Proteinuria, Creatinine) in Wistar Kyoto (WKY) rats and spontaneously hypertensive rats (SHR), without considering the type of drug treatment. The thickness of the lines is proportional to the level of significance and to the number of significant correlations between variables. The meaning of the schematic drawings is indicated in the left side of the scheme.

**Figure 6:**
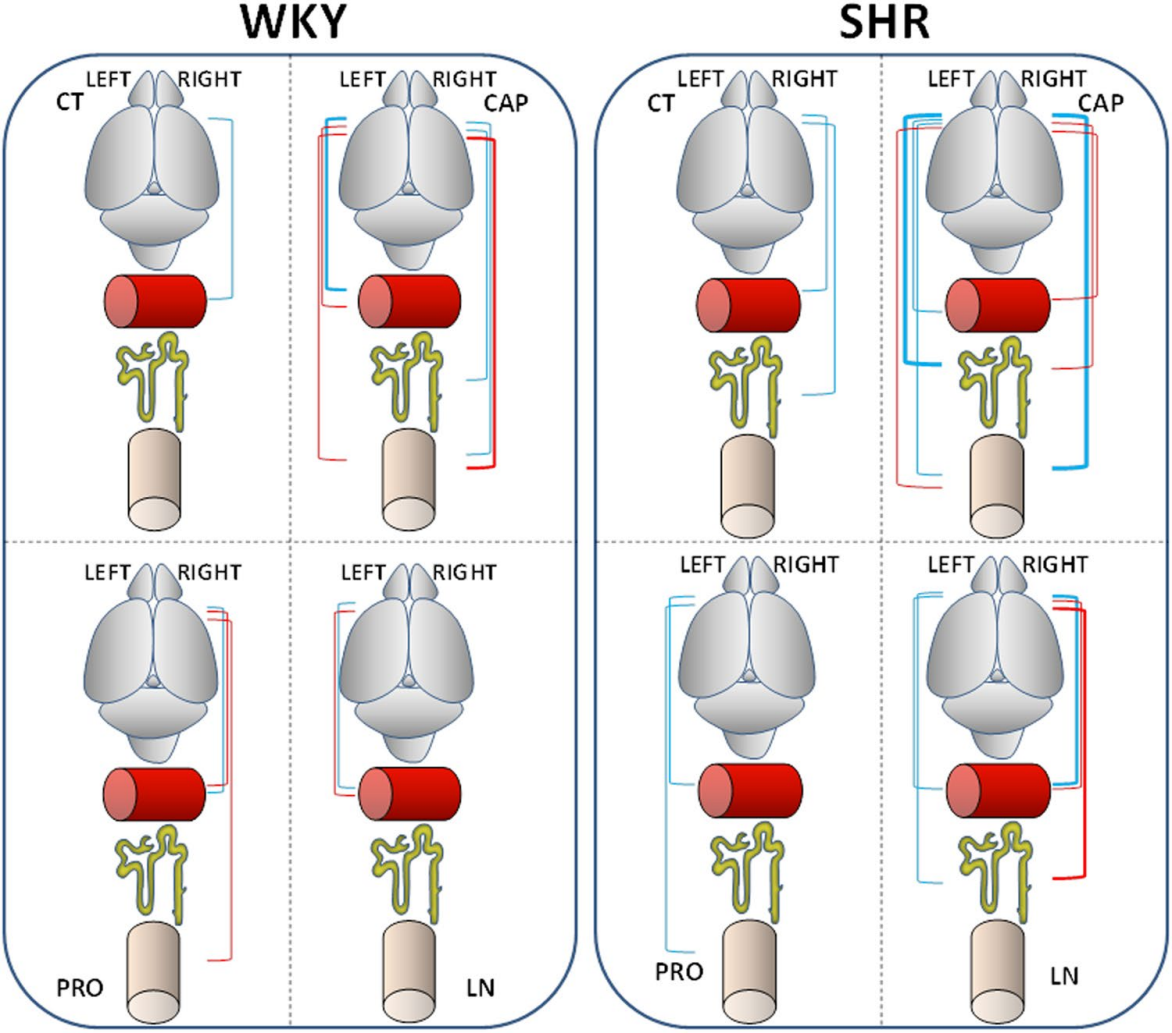
Schematic representation of the significant correlations between neuropeptidase activities of the left and right frontal cortex and plasma factors, physiologic parameters and urine metabolites in each of the four groups of Wistar Kyoto (WKY) rats and spontaneously hypertensive rats (SHR) depending on the type of treatment: Control (CT), captopril (CAP), propranolol (PRO), and L-NAME (LN). Positive correlations are in blue and negative in red. The thickness of lines is proportional to the level of significance and the number of significant correlations between variables. The meaning of the schematic drawings is shown in Fig. 5.

The results of the correlational study are shown in Tables 1, 2 and 3 and represented in Figs 5 and 6. Considering the values of the four groups (Table 1, Fig. 5), the comparison between Sol or MB neuropeptidase activities from the left or right frontal cortex versus the physiologic parameters or metabolic factors in plasma or urine of WKY rats or SHR demonstrated that the most correlations were found between neuropeptidase activities of the *left* frontal cortex in WKY rats. Most of these correlations were *positive* in nature and mainly with MB enzyme activities. However, within these correlations, a few *negative* correlations were observed with proteins and creatinine in urine (Table 1, Fig. 5).

**Table 1:** Left and right frontal cortex vs. physiologic and metabolic parameters regardless of drug treatment.

WKY						SHR					
LEFT			RIGHT			LEFT			RIGHT		
Correlation	r	p	Correlation	r	p	Correlation	r	p	Correlation	r	p
Sol AlaAP vs TCH	+0.495	0.003	Sol CysAP vs HDL	+0.402	0.02	***Sol AlaAP vs NO Ur***	−***0.481***	***0.005***	***Sol CysAP vs WB***	−***0.501***	***0.002***
Sol CysAP vs WI	+0.461	0.007	***Sol GluAP vs HDL***	−***0.511***	***0.002***	MB CysAP vs NO Ur	+0.469	0.006	***Sol GluAP vs WI***	−***0.409***	***0.02***
Sol CysAP vs DIU	+0.509	0.002	MB AlaAP vs WB	+0.475	0.005	MB CysAP vs Creat	+0.408	0.02	***Sol GluAP vs WB***	−***0.419***	***0.01***
Sol CysAP vs GLU	+0.621	0.0001				MB GluAP vs TCH	+0.453	0.009	MB CysAP vs GLU	+0.400	0.02
MB AlaAP vsWI	+0.470	0.006									
MB AlaAP vs DIU	+0.437	0.01									
MB AlaAP vs WB	+0.446	0.01									
MB AlaAP vs GLU	+0.675	<0.0001									
MB AlaAP vs NO Pl	+0.498	0.003									
***MB AlaAP vs Protur***	−***0.403***	***0.02***									
***MB AlaAP vs Creat***	−***0.433***	***0.01***									
MB CysAP vs WI	+0.438	0.01									
MB CysAP vs WB	+0.534	0.001									
MB CysAP vs GLU	+0.545	0.001									
MB CysAP vs NO Pl	+0.474	0.005									
***MB CysAP vs Creat***	−***0.404***	***0.02***									
MB GluAP vs WI	+0.612	0.0002									
MB GluAP vs DIU	+0.649	<0.0001									
MB GluAP vs GLU	+0.400	0.02									
***MB GluAP vs Creat***	−***0.428***	***0.01***									

Significant correlations between soluble (Sol) or membrane-bound (MB) aminopeptidase activities of the left or right frontal cortex with the different physiologic parameters or metabolic factors measured in plasma and urine in WKY rats and SHR, considering the data of the four groups all together. Pearson’s correlation coefficients (r) and p values are indicated. Negative correlations highlighted in italics. WI, water intake; DIU, diuresis; WB, water balance; TCH, total cholesterol; HDL, high-density lipoprotein; LDL, low-density lipoprotein; GLU, glucose; NO Pl, nitric oxide in plasma; NO Ur, nitric oxide in urine; Protur, proteinuria; Creat, creatinine.

**Table 2:** Left and right frontal cortex vs. physiologic and metabolic parameters in each group of WKY rats.

LEFT			RIGHT		
Correlation	r	p	Correlation	r	p
**CONTROL**					
No correlations			Sol GluAP vs HDL/LDL	+0.822	0.01
**CAPTOPRIL**					
Sol CysAP vs HDL/LDL	+0.963	0.0001	Sol GluAP vs Creat	+0.772	0.02
MB AlaAP vs HDL/LDL	+0.796	0.01	MB AlaAP vs WI	+0.776	0.02
***Sol CysAP vs LDL***	***−0.915***	***0.001***	***MB CysAP vs Protur***	***−0.788***	***0.01***
***MB AlaAP vs LDL***	***−0.798***	***0.01***	***MB CysAP vs Creat***	***−0.920***	***0.001***
***MB GluAP vs Creat***	***−0.898***	***0.002***	***MB GluAP vs Protur***	***−0.774***	***0.02***
***Sol CysAP vs NO Ur***	***−0.828***	***0.01***			
**PROPRANOLOL**					
			Sol GluAP vs LDL	+0.791	0.01
No correlations			***Sol CysAP vs LDL***	***−0.791***	***0.01***
			***MB AlaAP vs LDL***	***−0.762***	***0.02***
			***MB GluAP vs NO Ur***	***−0.770***	***0.02***
**L-NAME**					
Sol AlaAP vs TCH	+0.756	0.02	No correlations		
***MB GluAP vs HDL***	***−0.818***	***0.01***			

Significant correlations between soluble (Sol) and membrane-bound (MB) neuropeptidase activities of the left and right frontal cortex with the different physiologic parameters and metabolic factors measured in plasma and urine in WKY rats, considering each of the four drug treatment groups independently. Pearson’s correlation coefficients (r) and p values are indicated. Negative correlations highlighted in italics. WI, water intake; DIU, diuresis; WB, water balance; TCH, total cholesterol; HDL, high-density lipoprotein; LDL, low-density lipoprotein; NO Ur, nitric oxide in urine; Protur, proteinuria; Creat, creatinine.

**Table 3:** Left and right frontal cortex vs. physiologic and metabolic parameters in each group of SHR.

LEFT			RIGHT		
Correlation	r	p	Correlation	r	p
**CONTROL**					
			MB CysAP vs WB	+0.836	0.009
No correlations			MB AlaAP vs GLU	+0.801	0.01
			MB GluAP vs NO Pl	+0.773	0.02
**CAPTOPRIL**					
MB GluAP vs WB	+0.917	0.001	MB CysAP vs Protur	+0.946	0.0003
MB GluAP vs HDL	+0.796	0.01	Sol AlaAP vs NO Ur	+0.849	0.007
MB GluAP vs WI	+0.904	0.002	Sol GluAP vs NO Ur	+0.781	0.02
MB AlaAP vs DIU	+0.859	0.006	***Sol GluAP vs WB***	−***0.792***	***0.01***
Sol CysAP vs Creat	+0.781	0.02	***Sol CysAP vs GLU***	−***0.824***	***0.01***
***MB AlaAP vs Creat***	−***0.778***	***0.02***	***Sol GluAP vs GLU***	−***0.816***	***0.01***
***MB GluAP vs NO Ur***	−***0.911***	***0.001***	***Sol AlaAP vs WB***	−***0.758***	***0.02***
**PROPRANOLOL**					
MB GluAP vs TCH	+0.830	0.01	No correlations		
MB GluAP vs Creat	+0.862	0.005			
**L**−**NAME**					
MB AlaAP vs DIU	+0.912	0.001	Sol AlaAP vs TCH	+0.781	0.02
MB GluAP vs GLU	+0.896	0.002	Sol AlaAP vs LDL	+0.973	<0.0001
			***Sol CysAP vs WI***	−***0.892***	***0.002***
			***Sol GluAP vs WI***	−***0.879***	***0.004***
			***MB GluAP vs WI***	−***0.788***	***0.02***
			***Sol GluAP vs HDL***	−***0.757***	***0.02***
			***Sol AlaAP vs HDL/LDL***	−***0.826***	***0.01***
			***Sol GluAP vs HDL/LDL***	−***0.793***	***0.02***

Significant correlations between soluble (Sol) and membrane-bound (MB) aminopeptidase activities of the left and right frontal cortex with the different physiologic parameters and metabolic factors measured in plasma and urine in SHR, considering each of the four drug treatment groups independently. Pearson’s correlation coefficients (r) and p values are indicated. Negative correlations highlighted in italics. WI, water intake; DIU, diuresis; WB, water balance; TCH, total cholesterol; HDL, high-density lipoprotein; LDL, low-density lipoprotein; GLU, glucose; NO Pl, nitric oxide in plasma; NO Ur, nitric oxide in urine; Protur, proteinuria; Creat, creatinine.

Each strain of rats can be independently assessed depending on the type of treatment. In WKY rats (Table 2, Fig. 6), most of the correlations were observed in the CAP group with neuropeptidase activities from both the *left* and *right* frontal cortex. In the PRO group, only neuropeptidases from the *right* frontal cortex correlated with metabolic factors, mainly LDL. Few correlations were observed in the CT and LN groups. In SHR (Table 3, Fig. 6), while the CT and PRO groups exhibited few correlations, the CT group showed only correlations with the *right* frontal cortex whereas the PRO group showed correlations only with the *left* frontal cortex. More correlations were observed in the CAP group with both the *left* and *right* frontal cortex. In the LN group, the correlations were mostly with the *right* frontal cortex and *negative*.

## Discussion

In summary, the present results suggest (1) that while CAP treatment may have led to a bilateral connection between both the *left* and *right* frontal cortex with physiologic/metabolic parameters in both SHR and WKY rats, LN treatment induced a high number of correlations, mostly negative in nature, only with the *right* frontal cortex of SHR; (2) that vasoactive drug treatments may have modified the neuroendocrine connection by changing the predominance of the *left* or *right* frontal cortex in SHR and WKY rats; (3) that there are asymmetric neurovisceral connections, which may occur by the conversion of a neural impulse, involving the *left* or *right* frontal cortex, into secretion of different neuropeptidase-dependent relevant hormones that affect biological/metabolic parameters; (4) that neuropeptidases of the frontal cortex may play a role in regulating physiologic/metabolic parameters; and 5) that, considering the *left* and *right* behavior of aminopeptidase activities in the frontal cortex, CAP may have beneficial influence, whereas LN may have a deleterious one.

Previous results have revealed in frontal cortex specific asymmetrical patterns of some aminopeptidase activities in WKY that are altered in SHR^11^. These variations in asymmetries have been shown to be dependent on the enzymatic activity and the nature of the treatment. These results might be linked to cognitive alterations reported in these normotensive and hypertensive strains, which are affected by antihypertensive or hypertensive drug treatment. Particularly, there was a trend to vary the prevalence of the cortex side of enzymatic activity or to raise the right dominance in SHR compared to that in WKY rats and when the animals were treated with the hypertensive drug LN. As discussed below, the predisposition to right prevalence in SHR was suggested by the intra- and inter-hemispheric correlations observed between neuropeptidase activities in frontal cortex. Considering the bilateral behavior of neuropeptidases, a clear favorable effect of CAP and a detrimental of LN were observed. These results are interesting because modifications in intra- and inter-hemispheric relationships have been demonstrated to be involved in human^18^ and animal^19^ mood disorders. In addition, hypertension may cause mood alterations, which may be part of the production and development of diverse cardiovascular disorders, supporting a bidirectional connection between the cardiovascular system and brain. The purpose of the present research was to evaluate a possible lateralized interaction of certain neuropeptidase activities of the *left* or *right* frontal cortex versus various physiologic parameters or metabolic factors present in plasma or urine. The results clearly support such asymmetry in the brain that differs between normotensive and hypertensive subjects that is extended to virtually the entire organism.

The frontal cortex is related to cognitive functions and to the control of physiologic and metabolic processes^10^ through its connection with the hypothalamus^20^. Endogenous substrates for the analyzed neuropeptidases in the frontal cortex include oxytocin (CysAP), vasopressin (CysAP), enkephalins (AlaAP), angiotensins (AlaAP, GluAP) and cholecystokinin (GluAP)^21^ (Fig. 1). All these neuropeptides are involved in cognitive functions, hydroelectrolytic balance or cardiovascular control^10,22^. The present results reveal an asymmetric neurometabolic interactive relationship between the brain and physiologic functions reflected in plasma and urine factors.

**Figure 7:**
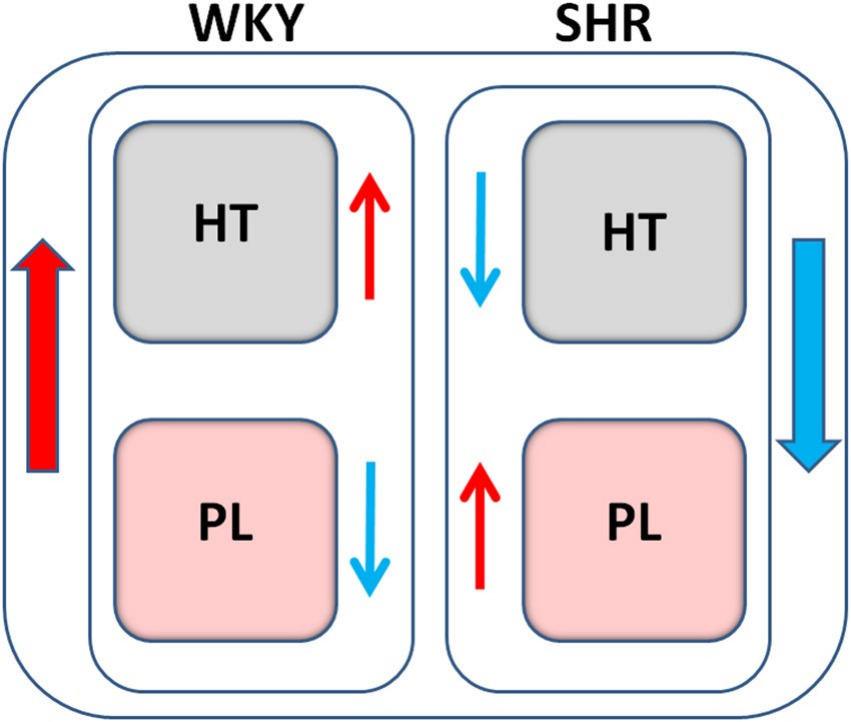
Schematic representation of the divergent response of aminopeptidase activities between the hypothalamus (HT) versus plasma (PL) of Wistar Kyoto (WKY) rats and spontaneously hypertensive rats (SHR) after treatment with propranolol. While the HT and PL response diverges between WKY rats and SHR, the HT and PL response also diverges within each strain^23^.

From the present results, several lines of evidence about systematic patterns of behavior could be highlighted and suggest, for instance, inverse relationships between the *left* versus the *right* sides of frontal cortex or between hypertensive (SHR) versus normotensive (WKY) rats. In this regard, considering the values of the four groups together, most correlations (Table 1, Fig. 5) were observed with the *left* frontal cortex of normotensive WKY rats, and most of these correlations were *positive* and associated with the MB fraction. This high number of correlations may be interpreted as a basal predominance of the left frontal cortex for functional or metabolic processes that selectively change under vasoactive drug treatments. However, all the *negative* correlations were obtained selectively between MB activities vs. creatinine and proteins in urine. In contrast, SHR exhibited a remarkable predominance of *negative* correlations between activities in the Sol fraction of the *right* frontal cortex and parameters of aqueous balance. These observations suggest a divergence between WKY rats and SHR: in WKY rats, the correlations were predominantly *positive* and between activities in the **MB** fraction of the *left* frontal cortex and aqueous balance parameters, whereas in SHR, the prevalent correlations were *negative* between activities in the **Sol** fraction of the *right* frontal cortex. Previous reports demonstrated an opposite behavior of aminopeptidase activities when hypothalamus versus plasma were compared in WKY or SHR and a divergent response when the hypothalamus and plasma were compared between WKY and SHR after PRO treatment (Fig. 7). This is supporting the association of the sympathetic nervous system in the regulation of these enzymatic activities^23^. The present results also suggest such divergence in the PRO group: while *negative* correlations with the *right* frontal cortex predominated in the WKY rats (without correlations with the *left* frontal cortex), SHR only demonstrated *positive* correlations with the *left* frontal cortex (without correlations with the *right*).

This divergent behavior is also highlighted by examining the results from another perspective: the correlations between the analyzed factors or the observed physiological parameters were established with specific neuropeptidases measured in samples collected from only one side of the frontal cortex, meaning that the same activity never correlated with the same factor on both sides. In instances when both the Sol and MB forms of the same enzyme correlated with the same parameter in the same group on both sides of the frontal cortex, the correlation was in the opposite direction, positive vs. negative, for the Sol and MB fractions. For example, whereas creatinine in the CAP group of WKY rats was *positively* correlated with **Sol** GluAP of the *right* frontal cortex, it was *negatively* correlated with **MB** GluAP of the *left* cortex (see Table 2). In SHR, WB in the CAP group was *negatively* correlated with **Sol** GluAP of the *right* cortex but *positively* correlated with **MB** GluAP of the *left* side. In addition, whereas **Sol** GluAP of the *right* frontal cortex was *positively* correlated with NO in urine, **MB** GluAP of the *left* side was *negatively* correlated (see Table 3).

**Figure 8:**
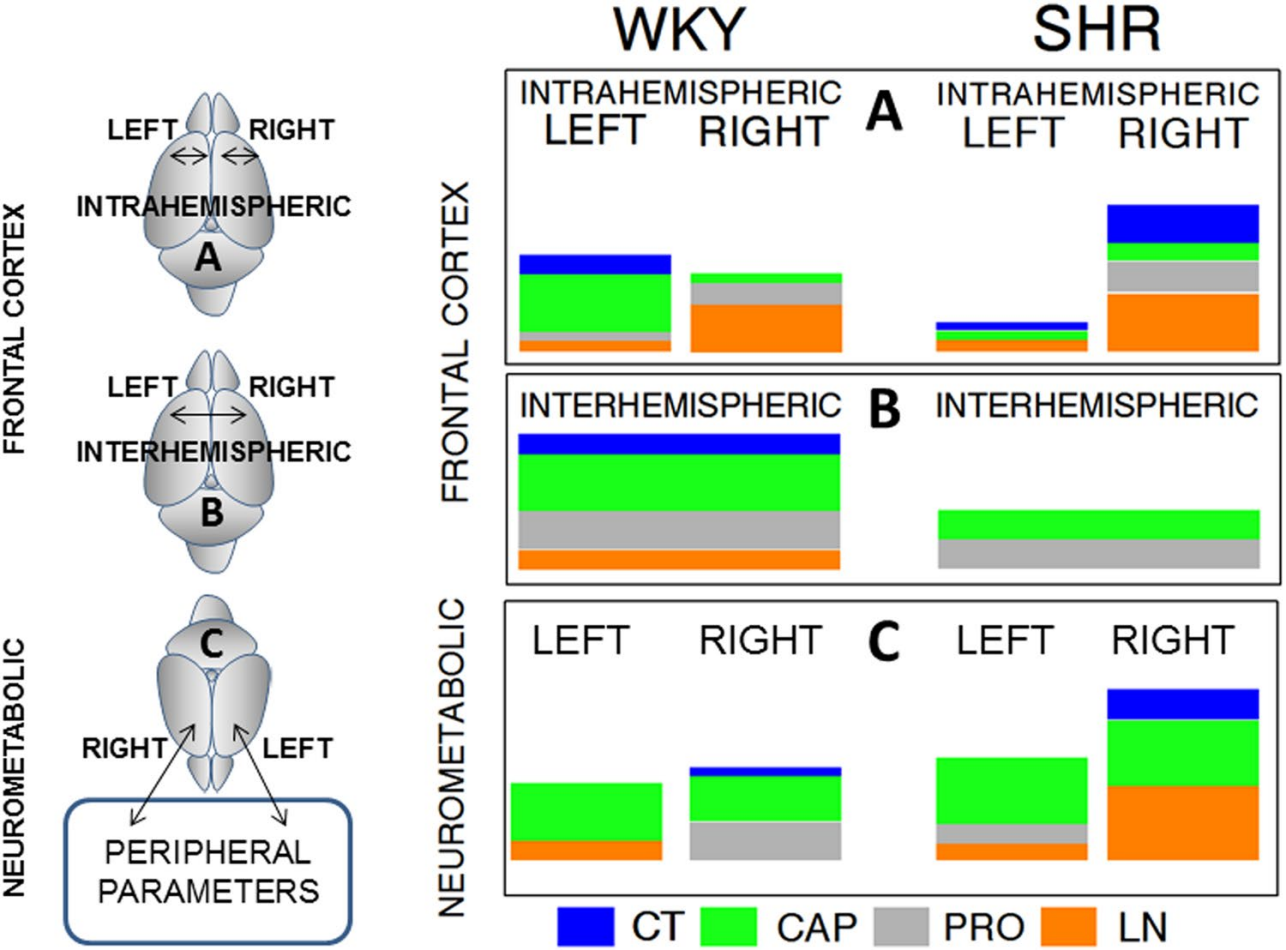
Comparative schematic representation of the total significant intrahemispheric (**A**) and interhemispheric (**B**) correlations previously obtained within the *left* or *right* frontal cortex (**A**) and between the *left* vs *right* frontal cortex (**B**)^11^ with the total correlations obtained between the *left* or *right* frontal cortex versus peripheral parameters (neurometabolic) (present results) (**C**) in the control (CT), captopril (CAP), propranolol (PRO) and L-NAME (LN) groups of Wistar Kyoto (WKY) rats and spontaneously hypertensive rats (SHR). The size of colored bars for CT, CAP, PRO or LN is proportional to the number of significant correlations obtained. Drawings of the left side of the figure illustrate the studied correlations. (**A**) Intrahemispheric correlations between neuropeptidase activities within the *left* or *right* frontal cortex^11^. (**B**) Interhemispheric correlations between the neuropeptidases of the *left* versus the neuropeptidases of the *right* frontal cortex^11^. (**C**) Significant correlations obtained between the *left* or *right* frontal cortex versus the peripheral functional, plasma or urine parameters.

Figure 8 comparatively analyzes the results of the correlations previously obtained within the frontal cortex^11^ with the correlations obtained between the cortex and the peripheral parameters (present results). In WKY rats, treatment with CAP decreased blood pressure levels and resulted in greater the intrahemispheric correlations between neuropeptidases in the *left* frontal cortex than in the *right* frontal cortex. In contrast, hypertension generated by LN treatment increased the intrahemispheric correlations on the *right* side. In SHR, high blood pressure levels (CT group) and increased blood pressure after treatment with LN (LN group) resulted in larger intrahemispheric correlations between neuropeptidases in the *right* frontal cortex than in the *left* frontal cortex. An examination of the interhemispheric correlations between neuropeptidases of the *left* and *right* frontal cortex in SHR and WKY rats reveals that the correlations did not appear in the untreated animals (SHR CT) and in those treated with LN. However, antihypertensive treatment (CAP and PRO) maintained the correlations, although to a lesser extent, indicating that hypertension decreases the interhemispheric correlation, whereas antihypertensive treatment maintains it. In the WKY rats, treatment with CAP favored the correlations between the peripheral parameters and neuropeptidases of both the *left* and *right* frontal cortex, while treatment with PRO enhanced correlations only with the *right* side. The CT and LN groups of WKY rats showed few or no correlations with both the *right* and *left* sides. In SHR, treatment with CAP also increased correlations with both the *left* and *right* frontal cortex. In the CT and LN groups of SHR, the correlations with the *right* side increased. These results suggest that hypertension and its increase with LN treatment induced a predominance of correlations with the *right* side, suggesting a beneficial influence of CAP and detrimental effect of LN.

Most of the aqueous balance parameters and urine metabolites correlated with the *left* or *right* frontal cortex in the CAP group of WKY rats and SHR (Tables 2 and 3). An increase in DIU after CAP treatment is a well-known effect, especially in WKY rats^24^. These results may be related to a previous study^14^, which suggested that in CAP-treated rats, CysAP, which acts mainly in the renal medulla, may regulate fluid balance. Regarding the connection of these parameters with brain asymmetry, Bianki *et al*.^25^ reported that both *right* or *left* hemisphere inactivation with potassium induced a similar response in water consumption. However, when animals were stronger motivated to drink, the *right* hemisphere predominated but when the motivation was weaker, the *left* hemisphere prevailed.

For lipids, the correlations were clearly asymmetric depending on the treatment and strain. In WKY rats, while correlations with the *left* side predominated in the CAP group, correlation with the *right* frontal cortex dominated in the group treated with PRO (Table 2). In SHR, most correlations with lipids were observed with the *right* side in the LN group (Table 3). No studies have associated brain asymmetry with lipid metabolism or hepatic function. Only Nikolaeva *et al*.^26,27^, as discussed below, reported a relationship between *left* or *right* handers and lipid metabolism. The liver connects with the hypothalamus through the autonomic nervous system^28^ and particularly with the frontal cortex^20^. The hypothalamus-liver connection also influences triglyceride metabolism through the sympathetic system^29^, which has also been shown to be related with the causes and development of metabolic syndrome^30^. The pattern of distribution of fatty acids in the frontal cortex is associated to the type of fat we have used in the diet and some of these fatty acids correlate significantly with certain aminopeptidase activities of the frontal cortex, suggesting not only a connection between peripheral tissues and the frontal cortex, presumably through the autonomic nervous system, but also that cognitive processes could be modified depending on the kind of fat with which we prepared the diet^31^.

Interestingly, GLU only correlated with the frontal cortex in SHR groups: *positively* with the *right* side in the CT group and with the *left* side in the LN group and *negatively* with the *right* frontal cortex in the CAP group (Table 3). LN treatment has been demonstrated to increase glycemia more in SHR than in WKY rats, probably due to the concomitant increase in insulin in WKY rats that is not present in SHR^32^, which is clearly supported with the present results (Fig. 4). Walters *et al*.^33^ have related high plasma GLU levels to alterations in the bilateral functioning of the frontal cortex, which in turn is associated with cognitive alterations.

**Figure 9:**
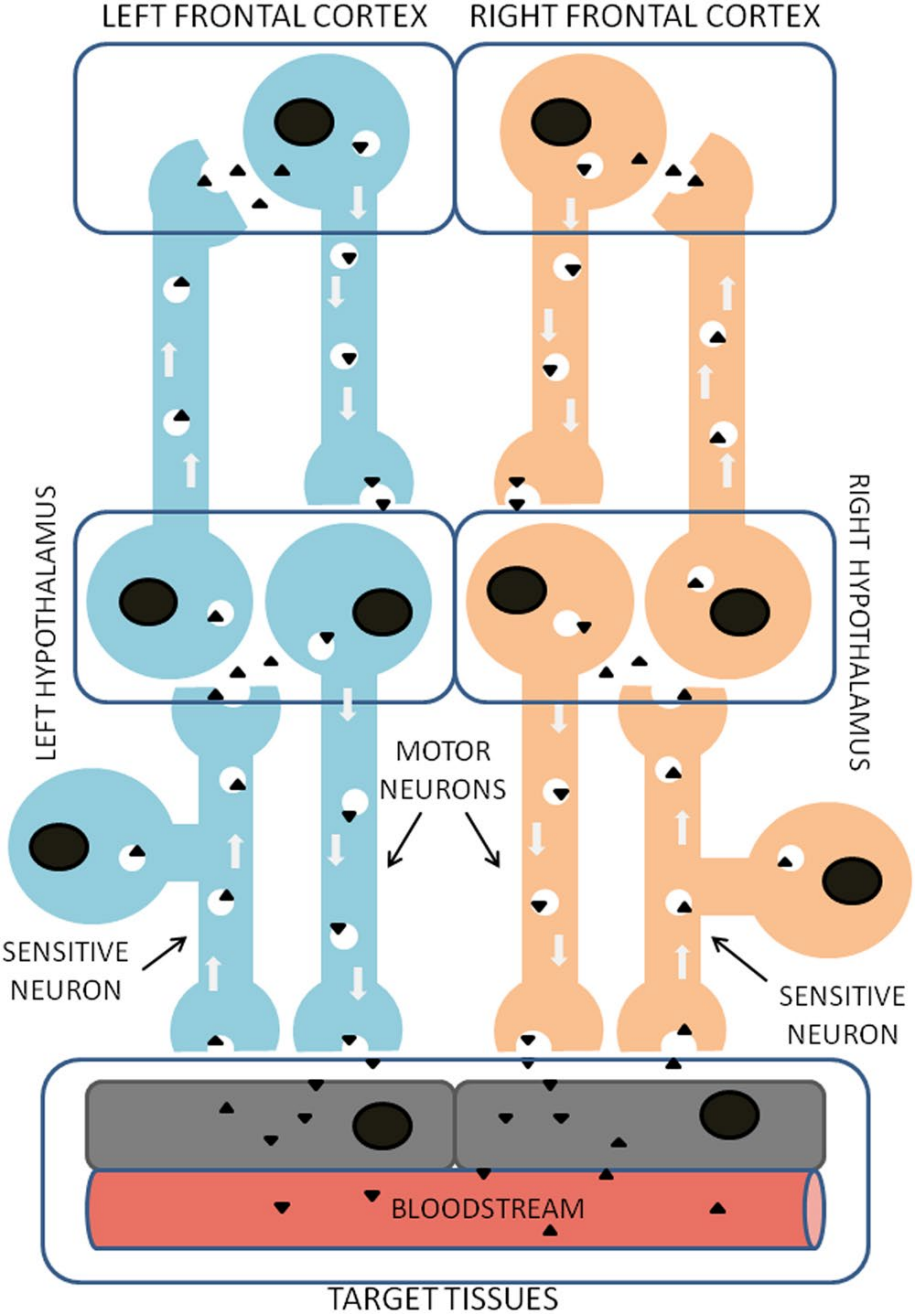
Illustrative scheme of the proposed mechanism for a possible functional and neurometabolic asymmetrical connection between the *left* and *right* frontal cortex and peripheral tissues through bidirectional pathways connecting frontal cortex and hypothalamus, as well as hypothalamus and target tissues through the autonomic nervous system, in which neuropeptidases (black triangles) would be involved.

Changes in the asymmetries of the frontal cortex can not only have cognitive consequences but they also affect peripheral and metabolic functions through the control that the *left* and *right* frontal cortex exert on the *left* and *right* hypothalamus and as a consequence on the autonomic nervous system (ANS). We have previously speculated on the mechanism of neurovisceral connection between hypothalamus, heart and kidney^34^ and demonstrated neurofunctional asymmetries that connect the activity of GluAP of the *left* or *right* frontal cortex with the *left* or *right* turning behavior of the rat^35^. Also, we showed neurometabolic asymmetries that connect a cerebral asymmetry in the content of dopamine with changes in plasma levels of GluAP and NO in the rat^36^. Similar asymmetry also affected heart function of rats^37^. All these consequences are presumably involving the ANS^34,36,37^. The asymmetric functioning of the frontal cortex can partly condition the asymmetric behavior of the ANS and consequently differentially determine the metabolic response of the organism and vice versa. Thus, the animal’s metabolic state can influence the lateralized functional predominance of the frontal cortex. Taking into account the reported connections between the frontal cortex and the hypothalamus^38^, considered as the main way to regulate autonomic and neuroendocrine responses^39^, we could hypothesize a lateralized bidirectional flow of neuropeptidases through axonal vesicular transportation between the frontal cortex and the hypothalamus, as well as between the latter and peripheral tissues, through the ANS (Fig. 9). Neuropeptidases would act by regulating their respective endogenous substrates (neuropeptides) centrally as well as in peripheral target tissues (peptides) and consequently, the physiological and/or metabolic functions that they could exert.

Considering the relationship: “increase in enzyme activity/decrease in the level of the susceptible substrates” and vice versa, the susceptible endogenous substrates of the enzymes analyzed (Fig. 1) may disclose, in general terms, the functional status of these substrates, revealing a predominance of the *left* or *right* frontal cortex, in connection with certain peripheral parameters. Particularly, focusing on specific results, we could suggest for example that in WKY, there was a clear increase in both sides of significant neurometabolic correlations after CAP treatment and a predominance biased to the *right* side with PRO treatment (Table 2). Specifically, for example, in the *left* frontal cortex, after CAP treatment there was an inverse correlation between CysAP and AlaAP with LDL (Table 2): the higher CysAP and AlaAP/the lower LDL and vice versa. This may mean that an increase of both activities in the *left* frontal cortex would be compatible with a decrease in ADH levels, a decrease in Ang III and an increase in Ang IV on the *left* side, which would be related to low plasma LDL levels. The opposite could be suggested with the decrease of both activities in the *left* frontal cortex. In SHR, the high frequency of significant correlations on both sides of the frontal cortex after CAP treatment is also observed and a shift towards the *right* predominance appears after L-NAME treatment. In particular, in the *left* frontal cortex, a highly significant positive correlation was observed between GluAP and AlaAP with water balance parameters (WI, DIU, WB) after CAP treatment (Table 3): the higher GluAP and AlaAP/the higher water balance parameters. This result may suggest that an increase of GluAP reflects decreased levels of Ang II together with increased Ang III and also that an increase of AlaAP involves a decrease of Ang III and an increase of Ang IV that would be directly related, as a consequence, with increased aqueous balance parameters (Table 3). Indeed, the neuropeptide Ang IV has been reported to be involved with water balance^40^. Therefore, these results could be well-matched with a significant role of Ang IV in the *left* frontal cortex in relation with aqueous balance after CAP treatment. In any case, further research would be necessary to conclusively be able to relate specific physiological functions or precise metabolic states with lateralization of neuropeptidases in the frontal cortex.

In conclusion, the present results describe for the first time asymmetric correlations between peripheral metabolic factors, physiologic processes and neuropeptidase activities in the frontal cortex. Such asymmetric changes occur depending on the strain and antihypertensive or hypertensive drug treatment. This observation definitively supports an asymmetric organization of neurovisceral integration. These results may be important in light of not only unexpected consequences of antihypertensive treatments but also of unilateral cerebral insults or pathologies such as Parkinson’s or Alzheimer’s diseases, schizophrenia or depressive disorders^7^, in which a unilateral origin and development, has clearly been established.

## Materials and Methods

### Study design

Thirty-two adult male WKY rats and thirty-two adult male SHR, weighing 100 to 150 g at the beginning of the experiments, were used in this study^11^. Each strain was divided into untreated CT (n = 8), CAP (n = 8), PRO (n = 8) and LN (n = 8) groups. CAP (Sigma, 100 mg/kg/day), PRO (Sigma, 100 mg/kg/day) and LN (Sigma 70 mg/kg/day) were added to the drinking water of the corresponding group during four weeks^11^. The dosages as well as the period of treatment were previously described as suitable to reach the entire action of the drugs^41,42^. To prevent the effect of circadian or seasonal variations, the experiments were performed in summer under environmental light conditions between 9:00 a.m. and 12:00 noon^7,11^. The study was carried out according to the European Communities Council Directive 86/609/EEC and received approval from the bioethics committee of the University of Jaén. To measure WI and DIU and to obtain urine samples, we singly housed rats in metabolic cages 24 h prior to sacrifice, and WB was calculated subtracting DIU from WI^14^.

### Surgical procedure, tissue collection and plasma or urine determinations

After four weeks of the corresponding treatments and after recording systolic blood pressure^11^, animals were anesthetized with equithensin (2 ml/kg body weight; 42.5 g/l chloral hydrate dissolved in 19.76 ml ethanol, 9.72 g/l Nembutal ©, 0.396 l/l propylenglycol, and 21.3 g/l magnesium sulfate in distilled water)^11^, and samples of blood were obtained from the left cardiac ventricle^8^. The animals were perfused with saline solution through the left cardiac ventricle and sacrificed^9^. The brain was quickly removed (less than 60 s), and the left and right frontal cortices were dissected^11^ according to the stereotaxic Paxinos and Watson atlas^43^. The selected area was between the anterior left or right border of the frontal lobe up to 13.2 mm anterior to the interaural line^11^. Brain samples were used to determine neuropeptidase activities in their soluble (Sol) and membrane-bound (MB) forms as described below^11^. Plasma was isolated by centrifugation of blood samples for 10 min at 2000 g using heparin as an anticoagulant and stored at −20 °C^8^. Total plasma cholesterol was determined colorimetrically using kits supplied by Sigma (St Louis, MO, 352-50)^44^. HDL cholesterol precipitated with phosphotungstic acid was quantified with the same method^44^. LDL cholesterol fractions were calculated using Friedewald’s formula^44^. NO levels in plasma and urine were analyzed as previously described^36^. Proteins and creatinine in urine and plasma GLU were determined colorimetrically using kits supplied by Spinreact (Girona, Spain).

### Enzymatic and protein assays

Enzymatic assays were performed as previously described^17^. To obtain the Sol fraction, we homogenized brain samples in a hypoosmolar medium (10 mM HCl-Tris buffer, pH 7.4) and ultracentrifuged them at 100,000 g for 30 min at 4 °C. The supernatants were used for Sol protein and enzyme assays. To obtain the particulate fraction, the pellets were rehomogenized in HCl-Tris buffer (pH 7.4) plus 1% Triton X-100 to solubilize MB proteins. After centrifugation (100,000 g, 30 min, 4 °C), the protein level and activity of MB enzymes were measured in triplicate in the supernatants. To ensure complete recovery of activity, we removed the detergent from the medium by adding adsorbent polymeric Bio-Beads SM-2 (Sigma) (100 mg/ml) and shaking the samples for 2 h at 4 °C. The activity of Sol and MB neuropeptidases, measured as GluAP, AlaAP and CysAP, was determined fluorometrically using the arylamide derivatives glutamyl-, alanyl- and cystinyl-β-naphthylamide as substrates as previously described^17^.

Briefly, GluAP levels were determined using Glu-β-naphthylamide as a substrate: 10 μl of each supernatant was incubated for 120 min at 37 °C with 1 ml of the substrate solution (2.72 mg/100 ml Glu-β-naphthylamide, 10 mg/100 ml bovine serum albumin (BSA), 10 mg/100 ml dithiothreitol (DTT) and 0.555 g/100 ml CaCl_2_ in 50 mmol/l HCl-Tris, pH 7.4). AlaAP and CysAP levels were measured using Ala- or Cys-β-naphthylamide as substrates, such that 10 µl of each supernatant was incubated for 30 min at 25 °C with 1 ml of the substrate solution, i.e., 2.14 mg/100 ml of Ala-β-naphthylamide or 5.53 mg/100 ml of Cys-β-naphthylamide, 10 mg/100 ml BSA, and 10 mg/ 100 ml DTT in 50 mM of phosphate buffer (pH 7.4 for AlaAP) and 50 mM HCl-Tris buffer (pH 6 for CysAP). The reactions were terminated by the addition of 1 ml of 0.1 mol/l of acetate buffer, pH 4.2. The amount of β-naphthylamine released as a result of the enzymatic activity was measured fluorometrically at a 412 nm emission wavelength with an excitation wavelength of 345 nm^17^. Proteins were quantified in triplicate using the Bradford method^45^ with BSA as a standard. Specific Sol or MB aminopeptidase activity was expressed as pmol of the corresponding substrate hydrolyzed per min per mg of protein. Fluorogenic assays were linear with respect to time of hydrolysis and protein content^17^.

### Statistical analysis

In order to reach the objectives of the study, we carried out three statistical analyses:

1. A descriptive analysis using count per group, mean and standard deviation for each covariate.
2. A three-factor ANOVA for each of the enzymatic activities analyzed with the following factors: (a) The factor group with four categories (CT, CAP, PRO and LN). (b) The factor hemisphere (with two categories: *left* and *right*) crossed with group. (c) The factor rat, with eight rats nested in each group and two measures for each rat in each hemisphere. The group and hemisphere factors were fixed-effect factors, and the factor rat was a random effect factor. The analysis was carried out using a multilevel mixed model^46^. The group-by-hemisphere interaction was the first test considered, and if significant (P < 0.10), then pairwise comparisons between hemispheres within groups and among groups within hemispheres were carried out using Tukey’s penalization because of the balanced sample sizes^47,48^. If the interaction was not significant, independent tests among groups and between hemispheres were carried out.
3. With other variates such as WI, DIU, WB, TCH, HDL, LDL, HDL/LDL, GLU, NO Pl, NO Ur, Proteinuria and Creatinine, a two factor ANOVA was carried out: a) the factor strain with two levels SHR and WKY and (b) the factor treatment with four categories (CT, CAP, PRO and LN). Because for all the variates the interaction was significant, comparisons of variate by variate in the second factor, using the classical t-test with Tukey´s penalization, were done.
4. Since the existence of correlations between enzymatic activities in the two hemispheres, a multivariate linear regression was used to study the correlation between the different covariates with each one of the enzymatic activities of the *left* and *right* frontal cortex. Because there was an induced variability by group, two analyses were carried out: (a) an unadjusted analysis of each covariate with the enzymatic activity in each hemisphere and (b) an adjusted analysis by group of each covariate with the enzymatic activity in each hemisphere. Corrections for the number of tests were not made because of the intrinsic exploratory nature of this analysis. Therefore, the obtained statistical significances should be taken with caution and be considered as suggestions.

All analyses were carried out with Stata 14.1.

## Data Availability

The datasets generated during and/or analysed during the current study are available from the corresponding author on reasonable request.
